# Celiac Disease and Dermatologic Manifestations: Many Skin Clue to Unfold Gluten-Sensitive Enteropathy

**DOI:** 10.1155/2012/952753

**Published:** 2012-05-30

**Authors:** Marzia Caproni, Veronica Bonciolini, Antonietta D'Errico, Emiliano Antiga, Paolo Fabbri

**Affiliations:** ^1^Division of Dermatology, Department of Medical and Surgical Critical Care, University of Florence, 50129 Florence, Italy; ^2^Department of Clinical Physiopathology, University of Florence, 50139 Florence, Italy

## Abstract

Cutaneous manifestations of intestinal diseases are increasingly reported both in the adult and in the children, and this association cannot longer be considered a simple random. Besides the well-known association between celiac disease (CD) and dermatitis herpetiformis (DH), considered as the cutaneous manifestation of gluten-dependent enteropathy, is more frequently reported also the association with other mucocutaneous diseases. Among these there are both autoimmune, allergic, and inflammatory diseases, but also a more heterogeneous group called miscellaneous. The knowledge about pathogenic, epidemiological, clinical, and diagnostic aspects of CD is increasing in recent years as well as those about DH, but some aspects still remain to be defined, in particular the possible pathogenetic mechanisms involved in the association between both CD and DH and CD and other immunological skin diseases. The aim of this paper is to describe the skin diseases frequently associated with CD, distinguishing them from those which have a relationship probably just coincidental.

## 1. Introduction

In recent years, the knowledge about pathogenic, epidemiological, clinical, and diagnostic aspects of celiac disease (CD) has rapidly increased.

CD, also known as celiac sprue or gluten-sensitive enteropathy, can be defined as a permanent intolerance to wheat gliadins and other cereal prolamins in the small bowel mucosa in genetically susceptible individuals. The main expression of the disorder consists in characteristic, though not specific, small intestine lesions that impair nutrient absorption and improve upon withdrawal of the responsible cereals. Nevertheless, the clinical presentation of the disease can often be misleading as highly variable from one patient to another, leading to frequent delays in diagnosis [1], thus it is important to take into account both the distinction between classical (typical), subclinical (atypical or mono-symptomatic), silent (asymptomatic) and potential/latent CD [2] as well as the extraintestinal manifestations of the disease and/or the different associated disorders affecting different organs and systems recently classified as autoimmune, idiopathic, chromosomal, and miscellaneous. Among them there are many mucocutaneous diseases. In 2006, Humbert et al. proposed to classify skin diseases associated with CD in those improved by gluten-free diet and those occasionally associated with CD, dividing them into four categories: autoimmune, allergic, inflammatory, and miscellaneous [3] (Table 1).

In the present paper, the main features of the skin and oral diseases with a proven association with CD and those that improve after a gluten free-diet were described. Moreover, other skin conditions sporadically associated with CD as well as dermatologic manifestation secondary to nutritional deficiencies due to the enteropathy were briefly reported.

## 2. Dermatitis Herpetiformis

The most important skin disease closely associated with CD is dermatitis herpetiformis (DH), currently considered as the cutaneous manifestation of gluten-dependent enteropathy. DH, initially described by Louis Duhring in 1983 [4], is considered an autoimmune skin disease with an estimated prevalence range from 1,2 to 39,2 per 100.000 and an incidence range of 0,4 to 2,6 per 100.000 per year with geographical variability. Males have a higher prevalence of DH [5]. In fact, most population-based studies to date have found male-to-female ratios ranging from 1,5 : 1 to 2 : 1 [6]. Interestingly, the opposite has been shown about gender prevalence of CD, with female-to-male ratios ranging from 2 : 1 to 4 : 1. The time of onset of the disease is variable. Cases of childhood DH are currently more often reported than in the past, but the average age at presentation varies from 30 to 40 years old [7, 8]. A recent epidemiological study conducted by Salmi et al. [9] in Finland reported some interesting results about the increasingly rarity of DH. Although the rates of incidence and prevalence of DH, in the Finnish population in the thirty years between 1980 and 2009, were higher than those of previous studies conducted elsewhere, in the course of time there was a downward trend especially in the 90s. In particular, the estimated prevalence rate was 75,3 per 100000, while annual incidence rates were respectively 5,2 per 100000 in 1980–1989, 2,9 per 100000 in 1990–1999 and 2,7 per 100000 in 2000–2009, with a decrease in incidence rate between the first and second 10-year period that was statistically significant. In the study of Salmi et al. [9] emerged a ratio between DH and CD of 1 : 8, that resulted lower than 1 : 5 showed in previous studies [10]. Theoretically, the risk of a celiac patient to develop DH remains high, but, being the diagnosis of the enteropathy and therefore the adoption of gluten-free diet ever earlier, the risk of DH is drastically reduced [9] as postulated also by Fry [11].

**Figure 1:**
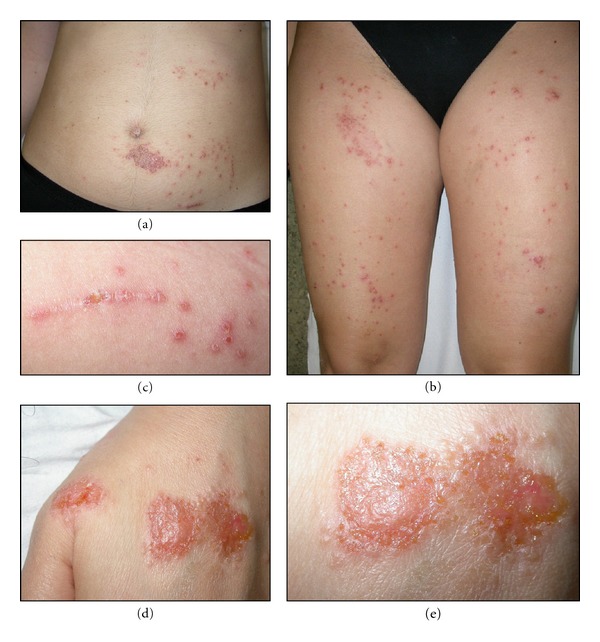
Erythematous, popular, and vesiculosus lesions in a patient with DH.

DH lesions show a typical polymorphism consisting of erythema, urticarial plaques, papules, grouped vesicles and blisters associated with intense itch and therefore followed by erosions, excoriations, and hyperpigmentation (Figure 1). In addition to the morphology, also the symmetrical distribution of the lesions on the extensor surfaces of the upper and lower extremities, elbows, knees, scalp, nuchal area, and buttocks is considered a hallmark of the disease. At times face and groins may be involved. DH is rarely observed in darker-skinned individuals [12, 13]; however, there were no significant clinical differences compared to those North European.

Since 1971, sporadic cases of DH presenting as palmoplantar purpura were reported. This uncommon skin manifestation is usually observed in the children, but a number of adult cases have been described. [14–16]

Since clinical presentation of DH is often atypical, especially in early and later stages in which prevailing scratching lesions, this diagnosis may not come to mind. DH must be differentiated from atopic dermatitis, scabies, papular urticaria, and impetigo in children, whereas eczema, other autoimmune blistering diseases (especially linear IgA bullous disease and bullous pemphigoid), prurigo nodularis, urticaria, and erythema multiforme should be considered in adults [17].

The diagnosis of DH is based on physical examination, histopathology, immunofluorescence studies, and serologic testing.

Routine histopathology of lesional skin of DH, that should ideally contain an intact vesicle or should be taken in the vicinity of early blisters [18], can be evocative, but not diagnostic, and nonspecific. Furthermore, the lesions present characteristic histopathological changes, in fact the initial inflammatory event is variable edema in the papillary dermis with discrete subepidermal vacuolar alteration and neutrophils along the dermal-epidermal junction. As the lesion develops, neutrophils, to a lesser extent eosinophils, and fibrin accumulate within the dermal papillae and form microabscesses. These become confluent resulting in a subepidermal blister. In early stages of the disease, the inflammatory infiltrate contains mostly neutrophils, but in later stages, variable numbers of eosinophils can be present [19]. However, a prevalent lymphocytic infiltrate was also reported by Warren et al. [20] probably corresponding to a later stage of the disease.

However, direct immunofluorescence (DIF) on perilesional skin should be considered the gold standard for the diagnosis [21, 22].

In particular, two different patterns of DIF are possible: (a) granular deposits in the dermal papillae and (b) granular deposits along the basement membrane. Sometimes, a combination of both patterns, consisting in granular IgA deposition along the basement membrane with accentuation at the tips of the dermal papillae, may be present [23, 24]. Recently Ko et al. suggested the existence of a third different pattern of IgA deposition at DIF, the fibrillar pattern, that may be related to a clinical variant of DH [25].

Also serologic tests, and in particular IgA antitissue transglutaminase antibodies (anti-tTG) and IgA endomysial autoantibodies (EMA), have become relatively sensitive and specific tools for detection of gluten-sensitive diseases and therefore of DH in subjects on a diet free. Other serologic tests for the diagnosis of DH include the detection of antibodies directed to epidermal TG (eTG), that is currently considered the key autoantigen in DH, as well as antideamidated gliadin peptides antibodies (IgA and IgG), that are particularly reliable in children under two years old, and antibodies against to the covalent complex tTG-deamidated gliadin peptides, that was coined as neoepitope [26, 27]. Currently, the diagnosis of CD in patients also affected by DH not requires further investigation because skin disease is sufficient for diagnosis of CD [28].

To date, the first-line therapy of DH, as well as CD, is gluten-free diet, that should not be considered as a mere symptomatic approach and therefore continue without interruption even after clinical remission [29]. Generally, several months are necessary to obtain the control of the skin disease. For this reason, other treatment may be used as symptomatic agents such as dapsone, sulfasalazine and sulphamethoxypyridazine, topical potent or very-potent corticosteroids, and antihistamines.

Since 1950, when the first report on successful use of dapsone in the treatment of DH was published [30], dapsone became the best tolerated symptomatic pharmacologic therapy for DH in both adults and children. In particular, the anti-inflammatory properties of this drug are linked to inhibition of neutrophil recruitment and local neutrophil- and eosinophil-mediated tissue injury.

Dapsone represents a valid therapeutic option during the 1- to 2-year period until the GFD is effective; dosages of 1/mg/kg/day can control itching and blister development. The commonest side effect of dapsone is haemolysis and patients should be seen within 2 weeks after starting the drug as haemolysis may be acute in some individuals [17].

Sulfasalazine and sulphamethoxypyridazine might provide an effective alternative to dapsone especially when it fails to control the disease or the therapy is complicated by adverse events [17].

## 3. Psoriasis

**Figure 2:**
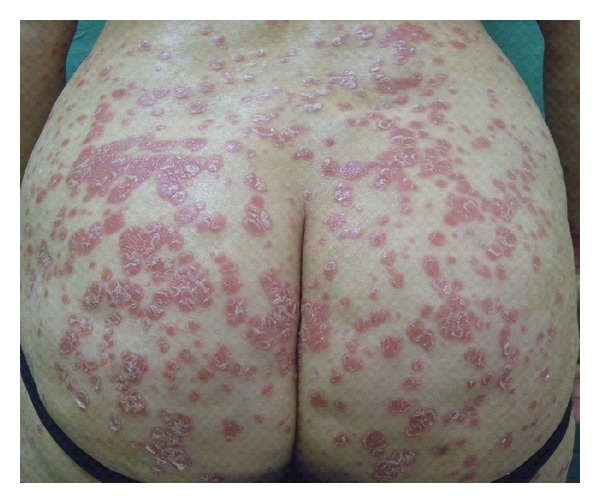
Erythematous scaly lesions of the buttocks in a patient affected by psoriasis.

Among the inflammatory skin diseases improved by gluten-free diet, psoriasis is one of the most important. Psoriasis is a common chronic relapsing inflammatory disease of the skin, which affects about 2% of general population and characterized by scaling, erythema, and less commonly postulation (Figure 2). Some patients have affected nails and joints (psoriatic arthritis) with an obvious decline in quality of life [31].

Psoriasis is an immunological disease with an important genetic predisposition linked to HLA-Cw*0602 [32], which is characterized by hyperproliferation of keratinocytes mediated by T cells [33]. In particular, Th1 and Th17 lymphocytes contribute to the pathogenesis of psoriasis through the release of inflammatory cytokines that promote further recruitment of immune cells, keratinocyte proliferation, and sustained inflammation. The inflammatory environment seems to be amplified due to the plasticity of T regulatory cells [34], that can convert into IL-17 producing cells. Moreover genetic, experimental and therapeutic evidences have highlighted a central role for the innate immune system in the pathogenesis of psoriasis [35].

The pivotal role of immune system in psoriasis pathophysiology is also confirmed by the frequent association with other immunological diseases.

The treatment of psoriasis is often difficult, although CD patients usually show an improvement only adopting the gluten-free diet, as stated above, and then suggesting pathogenetic differences compared to nonceliac-psoriatic patients. About the association between P and CD, we must consider a recent cohort study developed by Ludvingsson et al. that showed an increased risk of psoriasis both before and after CD diagnosis. Specifically, they showed that the absolute risk of future psoriasis in patients with CD was 135/100,000 person-years, with an excess risk of 57/100,000. The hazard ratio (HR) for psoriasis remained around 1.7 also when they excluded the first year of followup. Even 5 years after CD diagnosis we did detect more than 60% increased risk for psoriasis in patients with CD [36]. Several studies suggested a correlation between psoriasis and CD [37, 38], showing an improvement in psoriatic skin lesions after 3–6 months of gluten-free diet without other pharmacological approaches [39, 40]. However, at present the relationship between CD and psoriasis remains controversial since there are few data available in the literature, and this association is considered to be coincidental by some authors [41–43]. To our knowledge, no epidemiological studies are currently available demonstrating the prevalence of psoriasis in celiac patients. In 2001, Ojetti et al. showed a prevalence of CD of 4,34% in 92 psoriatic patients [37], while Zamani et al. denied the increase prevalence of CD in Iranian psoriatic patients with respect to general population as the estimated prevalence was 0,3% [44]. However, in 2009, a new study by Birkenfeld et al. confirmed the increased prevalence of CD also in Asian population affected by psoriasis with a prevalence rate varying from 0 to 29% against 0–11% of controls [45]. Finally, the most recent study of Montesu et al. showed a celiac prevalence of 2% in patients with psoriasis, confirming an increase than in the general population [46].

The mechanisms implicated in the possible association between CD and psoriasis, and consequently the effect of gluten-free diet on psoriatic skin lesions are currently not known. Three different hypotheses have been proposed:

1. abnormal small intestinal permeability, frequently present both in psoriatic [47] and in CD patients [48], could be a triggering factor between CD and psoriasis;
2. T cells play an important role in the pathogenesis of both psoriasis and CD. An increased number of T CD4+ cells in the blood, in the dermis, and in the epidermis of psoriatic patients have been documented [49]. In CD patients, gliadin induces a sensitization of T CD4+ cells [50], and this may play a role in the pathogenesis of psoriatic skin lesions [51];
3. psoriatic lesions in CD patients could be related to vitamin D deficiency, which is present both in CD [52] and in psoriasis [53, 54].

Moreover, recent observations of Troncone and Jabri [55]. suggested that psoriasis could be considered as a part of gluten sensitivity at least in a subgroup of patients. In those patients, the site of immunization against gluten may be extraintestinal and *⁄*or TG is probably not the main target antigen, since 16% of patients with psoriasis have been found to present high levels of IgA and *⁄*or IgG antibodies to gliadin in the absence of anti-TG antibodies, showing a significant reduction when they were put on a gluten-free diet [56].

## 4. Alopecia Areata

**Figure 3:**
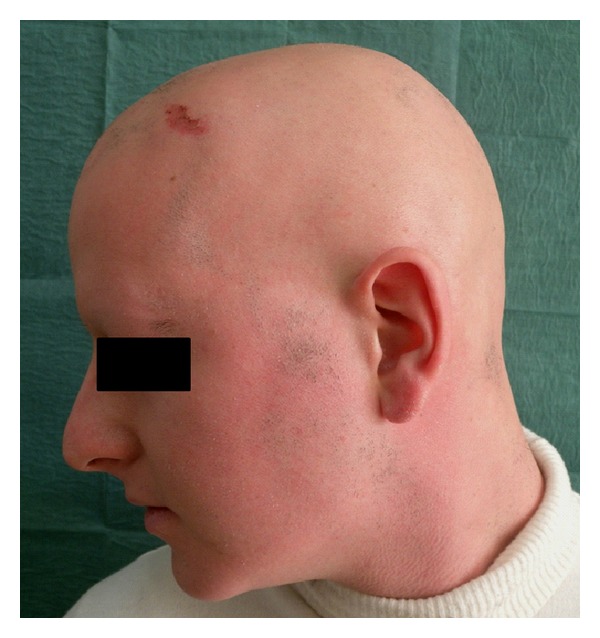
AA of scalp, beard, eyelashes, and eyebrows in patient affected by CD.

Alopecia areata (AA) is an autoimmune disease that presents as nonscarring hair loss, with a frequency ranging from 0.7% to 3.8% of their patients [57, 58]. Although some studies showed a significant male preponderance in adult age group, others demonstrated the opposite, indicating that AA likely affects males and females equally, as our personal clinical experience may suggest [59–61]. The disease prevalence peaks between the second and fourth decades of life [62], and pediatric reports are common accounting for 20% of all cases [63] (Figure 3).

For the first time, in 1995 Corazza et al. [64] described the association between AA and CD in 3 patients and developed a prospective screening program to ascertain whether this novel association could be real or coincidental. The estimated prevalence rate of CD in patients with AA was 1 in 85 [64], and therefore CD was included among the autoimmune diseases that may be associated with AA, in particular among those affecting the intestinal wall together with ulcerative colitis. By contrast, in 2008 Neuhausen et al. [65] considered the co-occurrence of CD and other autoimmune diseases both in celiac and their first-degree relatives in the North American population without finding an increased incidence of AA different from other autoimmune diseases such as insulin-dependent diabetes mellitus, juvenile rheumatoid arthritis/juvenile idiopathic arthritis, and hypothyroidism. Our review of the literature showed that the reported cases of association between these two conditions are few but, being often more severe variant of AA, in particular alopecia universalis, also as only clinical presentation of CD, an active search for CD using serological screening tests should be performed to diagnose the numerous cases of subclinical CD and avoid uncomfortable gastrointestinal and extraintestinal manifestations.

Although remission and recurrence may be observed during the clinical course of AA, many patients on gluten-free diet showed complete regrowth of scalp and other body hair and no further recurrence of AA at followup. The positive effects of gluten-free diet on the pattern of autoimmune conditions, such as AA, associated with CD have been attributed to a normalization of the immune response [66].

## 5. Chronic Urticaria

**Figure 4:**
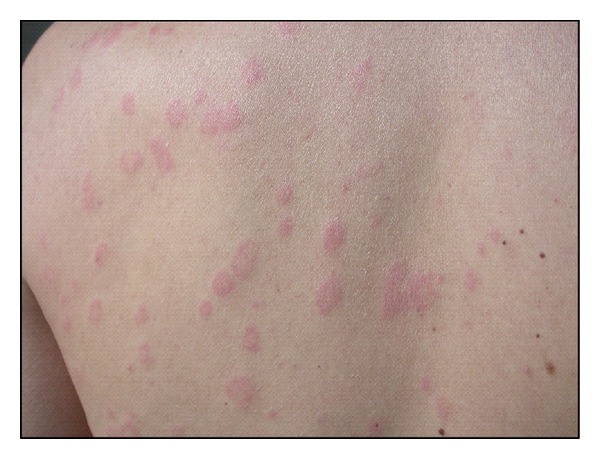
Pink-to-red edematous lesions, that have pale centers localized on the back of a patient affected by urticaria.

Urticaria is a common disorder, occurring in 15–25% of individuals at some point in life [67]. It is characterized by recurrent, itchy, pink-to-red edematous lesions that often have pale centers. The lesions can range in size from a few millimeters to several centimeters in diameter, and are often transient, lasting for less than 48 hours [68–71] (Figure 4). Approximately 40% of patients with urticaria also experience angioedema [68].

Urticaria is generally classified as acute (AU) or chronic (CU) depending on the duration of symptoms. AU refers to lesions that occur for less than 6 weeks, while CU to lesions that occur for more than 6 weeks; it is usually assumed that the lesions are present most days of the week [72]. Most cases of urticaria are acute; however, approximately 30% go on to become chronic. AU and CU are also distinguished by the prognosis, as AU can generally be easily managed and is associated with a good prognosis, while CU is often associated with significant morbidity and a diminished quality of life [70].

In 1987, Hautekeete et al. first described the association between CD and CU [71], although this is a matter still under debate [73]. Indeed, the relationship between the two diseases is not clear [74], but it can be speculated that autoimmunity induced by gliadin or by other unknown antigens may link CU and CD. The increased permeability of intestinal mucosa allows the passage of antigens that are responsible for CU pathogenesis by the formation of circulating immunocomplexes [75]. Both CD and urticaria are immune-mediated disorders, but they have a different pathogenesis. In fact, while CD is a Th1-mediated autoimmune response to gluten, urticaria could be supported by different mechanisms that range from Th2-driven response to allergens to Th1 autoimmunity [76]. In particular, autoimmune urticaria is related to autoantibodies against the *α* subunit of the high-affinity IgE receptor Fc*ε*R1 or against the *α* subunit of IgE. These antibodies are able to induce the mast cells degranulation and the consequent formation of anaphylatoxin [76].

However, the only epidemiologic study assessing the prevalence of CD in a population of adult idiopathic CU (ICU) patients was published in 2005 by Gabrielli et al. [73] without demonstrating an increased risk of CD in patients with ICU. These data, obtained on a population of 80 subjects affected by ICU and 264 healthy controls, were not confirmed by larger and more detailed epidemiological studies and are in contrast not only with several case reports, but also with the results of a recent study by Confino-Cohen et al. [77]. This study considered all autoimmune diseases potentially associated to CU finding thyroid diseases the most common one and also CD the more frequent among female affected by CU. In particular, when comparing women with CU with women in the control group, the odds of having CD was 57,8, and in most cases the diagnosis of CD followed that of CU, emphasizing that a screening through the determination of the serological markers of CD in patients suffering from CU may improve the prognosis of these patients.

Furthermore, even if no meta-analysis is still available, in some cases of CU the adoption of a gluten-free diet has proven effective in controlling the skin lesions [74, 76], further confirming that CU may be a cutaneous manifestations of CD and not only a chance association.

## 6. Hereditary Angioneurotic Edema

Hereditary angioneurotic edema (HANE) is a rare autosomal dominant genetic disorder resulting from an inherited deficiency or dysfunction of the C1 inhibitor, a plasma protease inhibitor that regulates several proinflammatory pathways. Three phenotypic variants of HANE have been defined: type I HANE, that is characterized by a quantitative and functional deficiency of C1 inhibitor (80–85% of cases); type II HANE, which is associated with normal C1 inhibitor levels, but low function (15–20% of cases); type III HANE, that includes rare cases, usually female, in which there are no alterations of quantity and functions of C1 inhibitor and the genetic defect in most cases involves the expression of factor XII (Hageman) resulting in increased production of bradykinin [78, 79].

Clinically, HANE is characterized by recurrent episodes of angioedema, without U or pruritus, which most often affect the skin or mucosal tissues of the gastrointestinal and upper respiratory tracts. Although generally benign conditions, laryngeal involvement can rapidly lead to fatal asphyxiation if left untreated. HANE usually presents in late childhood or adolescence in otherwise healthy subjects, and a familial history is present in approximately 75% of cases. These epidemiological features are useful for the differential diagnosis with acquired angioneurotic edema (AANE), which is not associated with a family history, and usually develops in older patients (fourth decade of life) with an underlying lymphoproliferative or autoimmune disease [80].

Cases of HANE associated with ulcerative colitis and Crohn's disease have been reported by Brickman et al. in 1986 [81] and after by Farkas et al. in 1999. In 2002, Farkas et al. first described the simultaneous occurrence of HANE and CD in a 14-year-old white male, which adopted gluten-free diet three years before following the diagnosis of CD, but represented similar clinical manifestations that was hardly ranked as HANE [82]. The knowledge and the ability to diagnose HANE is important not only for its frequent association with CD, particularly because of their confusion as Farkas et al. [83] reiterated in 2011. The aim of their study was to assess the prevalence of immunoregulatory disorders within the patient population affected by HANE, including CD, and contrary to other, CD was actually more common, with a prevalence of 3,1% in patients with HANE against that in healthy controls of 0,64%. Furthermore, according to the authors, similarities between the symptoms of HANE, and CD may cause difficulties in differential diagnosis, as well as in choosing the appropriate therapy, suggesting the screening for CD in HANE patients in whom abdominal attacks or neurological symptoms persist despite adequate management.

The classic activation pathway of the complement system plays a potential role in the immune regulation of both disorders, since C1 inhibitor is deficient in HANE and gluten is considered potent activator of the alternative pathway of the complement in CD [84]. Nevertheless, there might also be a genetically determined etiology of both diseases [85]. Complement testing is justified whenever the gastrointestinal symptoms of CD persist despite restoration of damaged mucous. Conversely, HANE unresponsive to adequate prophylaxis should prompt for complete gastrointestinal group tests [86].

In the literature, there are no data available about the effectiveness of the gluten-free diet.

## 7. Cutaneous Vasculitis

In the literature, there are sporadic reports about the association between cutaneous vasculitis (CV) and CD [86–88].

Vasculitis (V) is defined as inflammation directed at vessels, which compromises or destroys the vessel wall leading to haemorrhagic and/or ischaemic events. The skin is the most common involved organ, and clinical manifestations include U, infiltrative erythema, petechiae, purpura, purpuric papules, haemorrhagic vesicles and bullae, nodules, livedo racemosa, deep (punched out) ulcers, and digital gangrene. These varied morphologies are a direct reflection of size of the vessels and extent of the vascular bed affected, ranging from a V affecting few superficial, small vessels in petechial eruptions to extensive pan-dermal small-vessel V in haemorrhagic bullae to muscular vessel V in lower extremity nodules with livedo racemosa [89]. Aetiologically, vasculitis can be separated into primary V (idiopathic, including cutaneous leukocytoclastic angiitis, Wegener's granulomatosis, Churg-Strauss syndrome, and microscopic polyangiitis), secondary V (a manifestation of connective tissue diseases, infection, adverse drug eruption, or a paraneoplastic phenomenon), or incidental V (a histological finding that is the consequence of another pathological process such as traumatic ulceration or diffuse neutrophilic infiltrates) [90].

Some items may help to explain how so many different diseases can coexist, in fact leukocytoclastic V is often due to immunocomplex deposition on the vessel wall, and the antigen may be either exogenous or endogenous [91]. Therefore, increased intestinal permeability being present in CD, antigens can penetrate and form immunocomplexes, that can circulate because of the impaired phagocytic function of reticular endothelium system and be deposited in the skin [90]. Alternatively, an autoimmune sensitization may result because of the release of endogenous antigens from damaged small bowel mucosa [92].

Treatment of leukocytoclastic V is often difficult; however, the use of corticosteroids and mostly the adoption of gluten-free diet in patients with CD has proved of great help as reported also by Marsh and Stewart [90].

## 8. Atopic Dermatitis

Atopic dermatitis (AD) is a very common inflammatory skin disease in childhood, that has a large impact on the quality of life both of children and their families. In developed countries, AD is affecting 15–20% of the children [93, 94], and its cumulative incidence at the age of 6 based on the criteria of Hanifin and Rajka, determined in a recent population-based prospective birth cohort study in Denmark of 562 children, was 22.8% [95]. AD usually starts within the first 6 months of life. Remission during life occurs before the age of 15 years in 60–70% of cases, although some will relapse later. Most of the children have a family history of atopic diseases, and a high percentage of the children with AD are sensitized to food- and/or aero-allergens [96]. There is a large variability in the severity of the disease: most children have mild disease (70–84%) and are treated by general practitioners [97–99]. However, young age at onset (first year of life), coexistent respiratory allergy and urban living may be considered as factors of disease severity [100].

Genetic factors are thought to be involved in the development of AD involving several susceptibility loci.

The clinical manifestations of AD vary with age. It is often difficult to differentiate AD from other skin conditions such as scabies, contact dermatitis, seborrheic dermatitis, and also to those that we have already described among those more frequently associated with CD, such as DH and psoriasis [101].

As already mentioned above, CD is considered to arise from an inappropriate T-cell-mediated immune response against ingested gluten in genetically predisposed subjects [102] and therefore different from allergic, IgE-mediated reactions, in which the Th2-type lymphocytes are mostly involved [103]. Thus, one would hypothesize that Th1- and Th2-type immunity are present in a distinct patient population, but this is still a matter of controversy [101, 104]. In fact, some reports have suggested that allergy manifestations are more frequent in patients with CD [105], and asthma incidence is increased in celiac disease diagnosed in childhood [106]. Atopic disorders were more frequently found in children [107] and adult patients with CD and their relatives than in normal control subjects [108, 109]. Zauli et al. first showed that CD prevalence in Italian population of atopic patients was 1%, significantly higher than in general population [110]. On the contrary, one single case control study in children with CD denies the link between CD and allergy [111]. However, in 2004 Ciacci et al. considered both patients with and without malabsorption and showed that AD is about 3 times more frequent in patients with CD and 2 times more frequent in their relatives than in controls [112]. Unfortunately no data are available about efficacy of gluten-free diet in atopic patients with CD, because followup in the study conducted by Ciacci et al. was limited to 1 year and did not abate allergic manifestations, even if it cannot be excluded that a longer period of diet may have some effects [112].

## 9. Other CD-Associated Skin Conditions

As reported by Humbert et al. in 2006 [3], in addition to skin diseases with proven association with CD and those improved by gluten-free diet and/or with positivity of celiac serological markers, there are also fortuitous associations with other skin conditions. After a detailed review of the literature, we selected all the reported associations between CD and skin conditions. Although in none of these cases has been effectively demonstrated a pathogenetic link between the diseases, some of these associations are more common. Particularly lupus erythematosus [113], dermatomyositis [114], vitiligo [115], Behçet disease [116], linear IgA bullous dermatosis [117], and also both skin and mucosal manifestations of lichen [118, 119] are the most frequently reported, while prurigo nodularis [120], erythema nodosum [121], necrolytic migratory erythema [122], porphyria [123], cutaneous amyloidosis [124], pityriasis rubra pilaris [125], erythroderma [126], partial lipodystrophy [127], generalized acquired cutis laxa [128], ichthyosis [129], atypical mole syndrome, and congenital giant nevus [130] result very rare.

In addition to those listed above, there are also dermatological manifestations secondary to a deficiency of absorption of various nutrient in the intestine. The first and only case of pellagra associated with CD was reported in 1999 by Schattner [131], but CD patients may also present nonspecific dermatological disorders, that only a specialist can be traced to a specific vitamin or oligoelement. Therefore, in Table 2, we reported the main dermatological manifestations related to specific nutritional deficiencies, that a CD patient can develop during the course of the disease.

Finally, also oral cavity may be involved in course of CD by both dental disorders or oral mucosa manifestations. Recently, Rashid M et al. described oral and dental manifestations of CD, consisting in enamel defects, delayed eruption, recurrent aphthous ulcers, cheilitis, and atrophic glossitis and stressed that “the diagnosis of celiac disease can sometimes be made from a smile” [132].

## 10. Conclusion

Despite the knowledge about pathogenic, epidemiological, clinical and diagnostic aspects of CD is rapidly increased in the recent years, the possible mechanisms involved in the association with other diseases and in particular with the dermatological ones remain still unclear. Several hypotheses have been proposed depending on the type of the association, but the most probable may involve both a genetically conditioned lack of mechanisms for the maintenance of immunological tolerance, that consequently predisposes to autoimmunity and an abnormal small intestinal permeability, which may allow the crossing of endogenous or exogenous antigens and may provoke the immunological response, vascular alterations and, lastly, vitamin and aminoacid deficiency secondary to malabsorption in patients with CD.

Besides the importance of the diagnosis of DH, that is virtually always associated to CD and can be considered a specific marker of the disease, even the identification of the other dermatological conditions associated with gluten-sensitive enteropathy could be significant, highlighting the importance of a close collaboration between gastroenterologists and dermatologists. In fact, many skin diseases reported in this paper are actually more common in the celiacs or show atypical clinical presentation often associated with resistance to standard therapies in those patients. As a consequence, we suggest the screening for CD in patients affected by psoriasis, AA, CU, HANE, and AD, especially in cases resistant to first-line therapies.

## Figures and Tables

**Table 1:** Skin diseases associated with CD (adapted from Humbert et al. [3]. Gluten intolerance and skin diseases. Eur J Dermatol 2006; 16 : 4-11).

	Proved association	Improvement in skin disease by gluten free-diet or/and presence of serologic markers in several data	Fortuitous association (sporadic cases reports)
Autoimmune diseases	Dermatitis herpetiformis	Alopecia areata Cutaneous vasculitis	IgA linear dermatosis Dermatomyositis Vitiligo Lupus erythematosus Lichen sclerosous

Allergic diseases		Urticaria Atopic dermatitis	Prurigo nodularis

Inflammatory diseases		Psoriasis	Palmoplantar pustolosis Pytiriasis rubra pilaris Erythroderma

Miscellaneous diseases		Oral mucosa Chronic ulcerative stomatitis	Necrolytic migratory erythema Cutaneous amyloidosis Annular erythema Partial lipodystrophy Generalized acquired cutis laxa Ichthyosis Transverse leukonychia

**Table 2:** Dermatological manifestation secondary to nutritional deficiencies.

Zinc deficiency	Crusty-erythematous-squamous dermatitis localized to periorificial regions, genitals and flexures, associated with diffuse alopecia, stomatitis, balanitis, vulvar, and proctitis
Iron deficiency	Atrophy and dryness, itching, hair loss, atrophic glossitis, angular stomatitis, and koilonychia
Vitamin A deficiency	Pytiriasis rubra pilaris-like
Vitamin B12 and folic acid deficiency	Angular stomatitis, glossitis, and oral mucosa ulcers, hyperpigmentation
Vitamin PP deficiency	Pellagra
